# Hospital Worthies

**Published:** 1886-10-02

**Authors:** Jas. Goodchild Wakley


### HOSPITAL WORTHIES.

### DR. JAMES GOODCHILD WAKLEY.

We do not propose to give an account of the able and useful work the late Dr. Wakley did for the medical profession and the public as editor of *The Lancet* for the long period of twenty-five years. This aspect of his life has been fully dealt with by the medical press. There is, however, another field in which he has laboured which very closely concerns the readers of this Journal, and which has not heretofore been dwelt upon with the fulness which it merits. His will will no doubt be a surprise to many on this account, and because his devotion to the hospitals was little known or appreciated during his lifetime.

It is but right, therefore, now that Dr. Wakley has passed away, to emphasise his unvarying desire for the welfare of the hospitals. For very many years he has taken an increasing interest in these institutions. Three years ago, with this object, he had a pamphlet published and widely circulated, in the hope that the contributions on Hospital Sunday might thereby be greatly augmented. This failing, he determined, in the following year, to give £1,000 in the hope of stimulating others to go and do likewise. The letter in which this intimation was conveyed to the Lord Mayor was held by some to have been so worded as to call for criticism. The writer is in a position to know and to state, that Dr. Wakley absolutely declined to attach any condition to this gift, and that the desire of his heart was to make that gift as unostentatiously as possible, having in view the pressing pecuniary necessities of the hospitals and the need for others to be led in the same path of benevolence. This year Dr. Wakley gave another £1,000 to the Hospital Sunday Fund, and by the advice of those he consulted, though somewhat unwillingly, he attached certain conditions to this second donation, which he afterwards waived. In all this, and in every other instance where the hospitals have been concerned, Dr. Wakley always proved himself to be a determined supporter of free relief and free gifts to hospitals. Sickness and suffering know no distinction of sex or sect, and the late Dr. Wakley held, and held rightly, that, given a well-managed hospital, conducted upon the best known system, all its advantages should be freely given to the poor suffering sick, no matter whence they came or by what means they found their way to the hospital doors. Having been actively engaged with Dr. Wakley for many years past, the writer is speaking within his own knowledge when he says, that the late Editor of *The Lancet* was ever foremost in desiring to secure the utmost efficiency and the largest support for the hospitals, which the circumstances of the time or the needs of the suffering demanded and required.

The Hospitals Week. In all this Dr. Wakley followed in the footsteps of his father, whose connection with the Royal Free and other hospitals is too well known to need further reference here. But the last and most important aid which Dr. Wakley rendered to the hospitals demands the public recognition which a statement of the facts can alone secure. In the early part of the present year, although seriously ill, Dr. Wakley began to agitate in favour of a new and more strenuous effort in support of all the hospitals in connection with the Metropolitan Hospital Sunday Fund. He consulted a member of the Council more than once on the subject, and finally urged him to come to Heathlands and thresh the matter out. The gentleman in question urged pressure of other and absorbing duties and work, the desire which possessed him, as a junior member of the Council of the Hospital Fund, not to take any active steps personally this year, and several other reasons why he should be excused, but to little purpose, and in the end the Heathlands visit was arranged. The scheme known as "The Hospitals Week" was the outcome of this conference, and soon after, Dr. Wakley's illness took a most acute form. Other names became prominently associated with the scheme and its results, because the less Dr. Wakley was able to do, the more the other workers felt bound to exert themselves to make the movement, surrounded with unforeseen difficulties as it proved to be, the success it undoubtedly was. For, by reason of this new movement, more money was collected in the churches and chapels of the metropolis on Hospital Sunday, 1886, than ever before since the establishment of the Fund. Besides, as Mr. Michelli has proved from the facts he has published concerning its influence upon the income of St. Mary's Hospital, "The Hospitals Week" has directly brought considerable sums into the exchequer of many of the London hospitals. In these circumstances, Dr. Wakley's fellow-workers are anxious it should be known, in justice to the memory of a good man, that, had it not been for the late editor of *The Lancet*, no special effort would have been made for the London hospitals this year, and the satisfactory results recorded would not only have been wanting, but the hospitals must have received far less than usual from the Hospital Sunday Fund. Besides, it ought to be stated that the special supplements to *The Lancet*, in connection with the Hospitals Week, which did such yeoman's service, were produced at great cost, in accordance with Dr. Wakley's wishes, and that he carefully read over and considered the whole of them before publication. The debt the hospitals owe to Dr. Wakley is, therefore, as undoubted as it is considerable. By his death many lose a valued colleague and a most loyal and devoted friend. Words fail the writer when he tries to state in a sentence the feelings of gratitude and sorrow which have been caused by the death of so devoted and generous a friend of all the hospitals as the late Dr. Wakley undoubtedly was.

Dr. Wakley and "The Hospital." The conductors of THE HOSPITAL are also indebted to the late Dr. Wakley, for he was anxious to see this paper established. He promised to use in its favour any influence he possessed, and to co-operate to the fullest extent of his power to make THE HOSPITAL a genuine success by widening the public interest in hospitals, and so securing adequate funds for their efficient maintenance. In his later years especially, Dr. Wakley was imbued with the feeling expressed in Tennyson's line—

> "Men, my brothers, men the workers, ever
> reaping something new,"

for the benefit, consolation, and comfort of those who came within the sphere of his influence. Dr. Wakley was not, perhaps (he would certainly have been the last to claim or desire to be considered to be), a great man, as that word is commonly used in such a connection; but his endeavours to do good and to help forward those in sorrow or distress who could not help themselves, won the gratitude and love of the poor especially, and will cause his name to be remembered and cherished by the many.

> "Lives of good men all remind us
> We can make our lives sublime,
> And, departing, leave behind us
> Foot-prints on the sands of time."

